# Phenotypic characterization of carbapenem non-susceptible gram-negative bacilli isolated from clinical specimens

**DOI:** 10.1371/journal.pone.0256556

**Published:** 2021-12-02

**Authors:** Abera Abdeta, Adane Bitew, Surafel Fentaw, Estifanos Tsige, Dawit Assefa, Tadesse Lejisa, Yordanos Kefyalew, Eyasu Tigabu, Martin Evans

**Affiliations:** 1 National Clinical Bacteriology and Mycology Reference Laboratory, Ethiopian Public Health Institute, Addis Ababa, Ethiopia; 2 Department of Medical Laboratory Sciences, College of Health Sciences, Addis Ababa University, Addis Ababa, Ethiopia; 3 National Clinical Chemistry Reference Laboratory, Ethiopian Public Health Institute, Addis Ababa, Ethiopia; 4 Department of Applied Biology, School of Applied Natural Science, Adama Science and Technology University, Adama, Ethiopia; 5 Global One Health initiative, The Ohio State University, East African Regional Office, Addis Ababa, Ethiopia; 6 Laboratory Director and Microbiology Consultant, New York, New York, United States of America; Suez Canal University, EGYPT

## Abstract

**Background:**

Multidrug resistant, extremely drug-resistant, pan-drug resistant, carbapenem-resistant, and carbapenemase-producing gram-negative bacteria are becoming more common in health care settings and are posing a growing threat to public health.

**Objective:**

The study was aimed to detect and phenotypically characterize carbapenem no- susceptible gram-negative bacilli at the Ethiopian Public Health Institute.

**Materials and methods:**

A prospective cross-sectional study was conducted from June 30, 2019, to May 30, 2020, at the national reference laboratory of the Ethiopian Public Health Institute. Clinical samples were collected, inoculated, and incubated for each sample in accordance with standard protocol. Antimicrobial susceptibility testing was conducted using Kirby-Bauer disk diffusion method. Identification was done using the traditional biochemical method. Multidrug-resistant and extensively drug-resistant isolates were classified using a standardized definition established by the European Centre for Disease Prevention and Control and the United States Centers for Disease Prevention and Control. Gram-negative organisms with reduced susceptibility to carbapenem antibiotics were considered candidate carbapenemase producers and subjected to modified carbapenem inactivation and simplified carbapenem inactivation methods. Meropenem with EDTA was used to differentiate metallo-β-lactamase (MBL) from serine carbapenemase. Meropenem (MRP)/meropenem + phenylboronic acid (MBO) were used to differentiate *Klebsiella pneumoniae* carbapenemase (KPC) from other serine carbapenemase producing gram-negative organisms.

**Results:**

A total of 1,337 clinical specimens were analyzed, of which 429 gram-negative bacterial isolates were recovered. Out of 429 isolates, 319, 74, and 36 were Enterobacterales, *Acinetobacter* species, and *Pseudomonas aeruginosa* respectively. In our study, the prevalence of multidrug-resistant, extensively drug-resistant, carbapenemase-producing, and carbapenem nonsusceptible gram-negative bacilli were 45.2%, 7.7%, 5.4%, and 15.4% respectively. Out of 429 isolates, 66 demonstrated reduced susceptibility to the antibiotics meropenem and imipenem. These isolates were tested for carbapenemase production of which 34.8% (23/66) were carbapenemase producers. Out of 23 carbapenemase positive gram-negative bacteria, ten (10) and thirteen (13) were metallo-beta-lactamase and serine carbapenemase respectively. Three of 13 serine carbapenemase positive organisms were *Klebsiella pneumoniae* carbapenemase.

**Conclusion:**

This study revealed an alarming level of antimicrobial resistance (AMR), with a high prevalence of multidrug-resistant (MDR) and extremely drug-resistant, carbapenemase-producing gram-negative bacteria, particularly among intensive care unit patients at the health facility level. These findings point to a scenario in which clinical management of infected patients becomes increasingly difficult and necessitates the use of “last-resort” antimicrobials likely exacerbating the magnitude of the global AMR crisis. This mandates robust AMR monitoring and an infection prevention and control program.

## Introduction

The discovery of the antimicrobial agent is a fundamental milestone in the history of medicine and has saved millions of lives [1]. Antimicrobials were first used to treat infections in the 1940s [2]. Shortly after the discovery of antimicrobials, antimicrobial resistance emerged and with the recent increase in AMR, poses a serious threat to global public health [2].

The extensive use of antimicrobials for treating humans and animal infections along with globalization and international travel has led to the rapid spread of resistant strains [3]. The increasing incidence of healthcare-associated infections due to multidrug-resistant (MDR), extremely drug-resistant (XDR), and carbapenemase-producing gram-negative bacilli (GNB) has been widely reported [4–7].

The emergence and spread of multidrug-resistant gram-negative organisms (MDRO) pose serious threats to medical services and patient outcomes. Infections caused by carbapenemase-producing and carbapenem resistant Enterobacterales, *Acinetobacter baumannii*, *Pseudomonas aeruginosa* result in increased patient morbidity and mortality. This can result in significant additional health-care costs for patient management as well as outbreak control [8–11].

Carbapenem-resistant and carbapenemase producing organisms such as *E*. *coli*, *K*. *pneumoniae*, *Acinetobacter* species and *P*. *aeruginosa* have become one of the most important causes of nosocomial and community-acquired infections. They can cause urinary tract, respiratory tract, bloodstream, meningitis, malignant external otitis, intra-abdominal infection and wound infections. Some of the important virulence factors that are implicated in these isolates include capsular polysaccharides, lipopolysaccharides, fimbrial adhesins, siderophores, efflux pumps, hemolytic factors, iron acquisition system survival and immune evasion during infection [8–16].

Multidrug resistance has increased globally and is a major public health threat. Recent investigations reported the emergence of multidrug-resistant bacterial pathogens from different origins including humans, poultry, cattle, and fish that increase the need for routine application of antimicrobial susceptibility testing (AST) to select the antibiotic of choice as well as screening of emerging MDR strains [17–24].

International subject matter experts came together through a joint initiative by the European Centre for Disease Prevention and Control (ECDC) and the United States Centers for Disease Control and Prevention (CDC), to create a standardized international definition with which to describe acquired resistance profiles [25].

MDR is defined as acquired nonsusceptibility to at least one agent in three or more antimicrobial categories, XDR as nonsusceptibility to at least one agent in all but two or fewer antimicrobial categories (i.e., bacterial isolates remain susceptible to only one or two categories), and PDR (Pandrug-resistant) as nonsusceptibility to all agents in all antimicrobial categories. To apply these definitions, bacterial isolates should be tested against all or nearly all of the antimicrobial agents within the antimicrobial categories and selective reporting and suppression of results should be avoided [25].

The common mechanism of developing resistance to carbapenem antibiotics is through carbapenemase enzyme production [26]. Carbapenemase is the most versatile family of β- lactamases and recognizes almost all hydrolysable β-lactams, and most are resilient against inhibition by all available β-lactamase inhibitors [26]. *Klebsiella pneumonia* carbapenemase (KPC) hydrolyzes penicillin, all cephalosporins, monobactams, carbapenems, and β-lactamase inhibitors. Metallo-β-lactamases usually exhibit resistance to penicillin, cephalosporins, carbapenems, and the clinically available β-lactamase inhibitors but are inhibited by monobactams [27].

There is a considerable knowledge gap regarding risk factors associated with the occurrence of MDR bacteremia [28]. Identifying risk factors for acquiring gram-negative bacteremia could potentially help patient care and management [28].

Despite the increasing global burden of multidrug resistance and carbapenemase-producing gram-negative bacilli, data on multidrug resistance and carbapenemase-producing gram-negative bacilli in Ethiopia is scarce. As a result, the objective of this study was to determine the prevalence of MDR, XDR, carbapenem non-susceptible and carbapenemase-producing gram-negative bacilli from various clinical specimens and to phenotypically characterize carbapenem non-susceptible isolates.

## Materials and methods

### Study design, site, and period

A prospective cross-sectional study was conducted from June 30, 2019, to May 30, 2020, at the National Clinical Bacteriology and Mycology Reference Laboratory on clinical samples collected the NRL and referred from different healthcare settings in Addis Ababa.

### Sample collection and processing

Microbiological specimens from body fluids, ear swabs, sputum, urine, pus, cerebrospinal fluid, blood, and tracheal aspirates were processed following standard procedures [29]. Appropriate transport media were used in case of sample transportation delays. A total of 1,337 clinical specimens were collected during the study period. Specimens collected from each patient were inoculated onto culture media and incubated at appropriate temperatures and periods according to standard protocols related to each sample [29]. Identification was done using the conventional biochemical method [29]. Gram staining, colony characterization, and biochemical tests were conducted as part of the identification process. AST was done by the Kirby Baur disk diffusion method as per CLSI M100 2020. All frequently isolated Enterobacterales, *Acinetobacter* species, and *P*. *aeruginosa* recovered from the various clinical specimens during the study period were included. Necessary variables such as socio-demographics (age and sex), specimen type, referring health facilities, patient location, and previous antibiotic exposure from the test request form were entered onto pre-configured WHONET software version 2019.

### Bacterial isolation, identification and antimicrobial susceptibility testing

#### Bacterial isolation and identification

The specimens were inoculated onto appropriate culture media, incubated at appropriate temperature and time following standard procedure [29]. The growth was inspected to identify the bacteria. Initial identification of bacteria was done based on Gram reaction and colonial morphology. Gram-negative rods were identified by performing a series of traditional biochemical enzymatic and carbohydrate fermentative tests on triple sugar iron agar (Liofilchem, Roseto degli Abruzzi, Italy), oxidase strips (Liofilchem, Roseto degli Abruzzi, Italy), Simon’s citrate agar (Liofilchem, Roseto degli Abruzzi, Italy), and lysine iron agar (Liofilchem, Roseto degli Abruzzi, Italy). Indole production and motility were obtained using sulfide-indole-motility (SIM) medium (Liofilchem, Roseto degli Abruzzi, Italy). Urease production was obtained using a urea agar base supplemented with 40% urea solution (Oxoid Ltd., Basingstoke, Hampshire, England).

#### Antimicrobial susceptibility testing

The Kirby Bauer disk diffusion method was used with Muller Hinton agar (Oxoid Ltd. Basingstoke, Hampshire, England) to determine antimicrobial susceptibility patterns of the isolates and CLSI M100 2020 was used to interpret the results [30].

The following antimicrobial discs were used: ampicillin (10μl), amoxicillin/clavulanic acid (20/10μl), piperacillin/tazobactam (100/10μl), cefazolin (30μl), cefuroxime (30μl), ceftazidime (30μl) obtained from Hardy Diagnostics, Santa Maria, CA, USA. Ceftriaxone (30μl), cefotaxime (30μl), cefepime (30μl), imipenem (10μl), meropenem (10μl), amikacin (30μl), gentamicin (10μl), and tobramycin(10μl) were obtained from OXOID LTD., Basingstoke, Hampshire, England. Nalidixic acid(30μl), ciprofloxacin(5μl), trimethoprim/sulfamethoxazole (1.25/23.75μl), nitrofurantoin(300μl), and tetracycline(30μl) from Liofilchem, Roseto degli Abruzzi, Italy.

### Detection of carbapenemase

The Clinical and Laboratory Standards Institute CLSI (2010) introduced the modified Hodge test for carbapenemase detection, but this method can only be used for the accurate detection of KPC-type carbapenemase in *Enterobacterales* [31]. CLSI (2012) recommended the Carba NP test method for the detection of carbapenemase in gram-negative bacilli; however, the preparation of the reagents required for this test is complicated and the solutions cannot be stored for extended periods, limiting its clinical application [32].

In 2015, the carbapenem inactivation method (CIM) which is easy to operate and highly sensitive for the detection of carbapenemase was introduced [33]. In 2017, based on the CIM method, CLSI recommended the modified carbapenem inactivation method (mCIM), However, it is a relatively complex method and can only be used to detect carbapenemase in *Enterobacterales* and *P*. *aeruginosa* [34]. In 2018, based on the mCIM, a simplified carbapenem inactivation method (sCIM) was designed for accurate detection of carbapenemase in gram-negative bacilli [35].

#### Modified carbapenem inactivation method

In the mCIM, a 1 mL loopful of *Enterobacterales* or a 10 mL loopful of *P*. *aeruginosa* from blood agar (Oxoid Ltd., Basingstoke, Hampshire, England) plates was emulsified in 2 mL trypticase soy broth (TSB) (Oxoid, Ltd., Basingstoke, Hampshire, England). A meropenem (10μl) disk (Oxoid Ltd., Basingstoke, Hampshire, England) was then immersed in the suspension and incubated for a minimum of 4 h at 35°C. A 0.5 McFarland suspension of *E*. *coli* ATCC 25922 was prepared in saline using the direct colony suspension method. A Mueller Hinton agar (MHA) (Oxoid Ltd., Basingstoke, Hampshire, England) plate was inoculated with *E*. *coli* ATCC 25922 using the routine disk diffusion procedure. The meropenem disk was removed from the TSB and placed on a MHA plate previously inoculated with the *E*. *coli* ATCC 25922 indicator strain. Plates were incubated at 35°C in ambient air for 18–24 h. An inhibition zone diameter of 6–15 mm or colonies within a 16–18 mm zone was considered to be a positive result, and a zone of inhibition of ≥19 mm was considered to be a negative result [30].

#### Simplified carbapenem inactivation method

The sCIM is based on the mCIM with an improved procedure. Instead of incubating the antimicrobial disk in the organism culture media for 4 hours as in the mCIM, the organism to be tested was smeared directly onto an antimicrobial disk in the sCIM. To perform the sCIM for *Acinetobacter* species, a 0.5 McFarland standard suspension (using direct colony suspension method) of *E*. *coli* ATCC 25922 was diluted 1:10 in saline and inoculated onto the MHA plate, following the routine disk diffusion procedure. Plates were allowed to dry for 3–10 min [35].

Subsequently, 1–3 colonies of the test organisms grown overnight on blood agar were smeared onto one side of an imipenem disk (10μg); immediately afterward, the side of the disk having bacteria was placed on the MHA plate previously inoculated with *E*. *coli* ATCC 25922. The imipenem disk placed on an MHA plate was used as the control [35].

All plates were incubated at 35°C for 16–18 hours in ambient air. Bacterial strains that produced carbapenemase hydrolyze imipenem; hence the susceptible indicator strain grows unchecked. If the zone of inhibition around the disk gave a diameter of 6–20 mm, or the satellite growth of colonies of *E*. *coli* ATCC 25922 around the disk a zone diameter of ≤ 22 mm, the result was considered carbapenemase positive; a zone of inhibition ≥ 26 mm was considered to be a negative result; a zone of inhibition of 23–25 mm was considered to be a carbapenemase indeterminate result [35].

#### Differentiation of metallo-β-lactamase from serine carbapenemases

The modified carbapenem inactivation method positive Enterobacterales (formerly *Enterobacteriaceae*) and *P*. *aeruginosa*, and the Simplified Carbapenem Inactivation Method positive *Acinetobacter* species detected were further screened for Class B metallo-carbapenemase (MBLs) which are characterized by inhibition with metal chelators like EDTA. Meropenem disks containing EDTA (Liofilchem, Roseto degli Abruzzi, Italy) were used to differentiate metallo-β-lactamase and serine carbapenemase. A ≥ 5-mm increase in zone diameter for eCIM vs. zone diameter for mCIM was considered MBL positive. A ≤4mm increase in zone diameter for the eCIM vs zone diameter of mCIM was considered MBL negative. Carbapenemase positive, metallo carbapenemase negative, gram-negative bacilli were considered serine carbapenemase producers [30].

Quality control recommendations: *K*. *pneumoniae* ATCC® BAA-1705™ *E*. *coli* ATCC® 25922™ were used as positive and negative controls respectively for meropenem with EDTA [30].

#### Differentiation of *Klebsiella pneumoniae* carbapenemase (KPC) from other serine carbapenemases

Serine carbapenemase producers were further screened for *Klebsiella pneumoniae* carbapenemase (KPC). Minimum inhibitory concentration (MIC) ug/mL KPC strips consisting of meropenem (MRP)/meropenem + phenylboronic acid (MBO) (Liofilchem, Roseto degli Abruzzi, Italy) were used to detect *Klebsiella pneumoniae* carbapenemase (KPC) producing gram-negative isolates [36].

Well isolated colonies from an overnight blood agar plate were suspended in saline to achieve a 0.5 McFarland standard turbidity (1.0 McFarland if mucoid) to obtain a confluent lawn of growth after incubation. The strip was applied to the agar surface with the scale facing upwards and the code of the strip to the outside of the plate. The agar plates were incubated in an inverted position at 35 ± 2°C for 16–20 hours in an ambient atmosphere. The incubation time was extended to 48 hours to capture any slow-growing gram-negative non-fermenters [36].

*Result interpretation*. The ratio of MRP/MBO of ≥8 or ≥3 log2 dilutions was interpreted as a KPC producer. The phantom zone or deformation of the ellipse was interpreted as positive for KPC regardless of the MRP/MBO ratio [36].

*Quality control recommendations*. *K*. *pneumoniae* ATCC® BAA-1705 (intrinsic KPC production) and *E*. *coli* ATCC® 25922 and were used as positive and negative controls respectively to check the reactivity of KPC strips [36].

### Reagent quality assurance

The quality of culture media, antimicrobial disks, and gradient strips were checked as per CLSI and EUCAST guidelines, laboratory SOPs, and the manufacturer’s instructions as applicable.

### Data analysis and interpretation

The WHONET 2019 version was used to enter, clean, and analyze the data. The risk factors for MDR gram negative bacilli acquisition were examined by exporting WHONET data to SPSS statistics version 23. Tables and figures were used to present the results. Chi-square and univariate analysis were used to determine the association between multidrug-resistant gram-negative bacilli and different risk factors. P-values less than 0.05 were considered statistically significant.

### Ethical considerations

The study was conducted after ethical clearance was obtained from the Department Research and Ethical Review Committee of the Department of Medical Laboratory Sciences, College of Health Sciences, Addis Ababa University. Official permission from the Ethiopian Public Health Institute was obtained. All results were kept confidential; the patient’s name and other personal identifiers were encrypted and the sample identifier automatically generated by the laboratory information system (LIS-Polytech) was used.

## Results

During the study period, 1,337 specimens were analyzed providing 429 gram-negative isolates. Of these 293 were Enterobacterales, 74 were *Acinetobacter* species, and 36 were *P*. *aeruginosa*. The number of samples based on specimen types were as follows: blood (364), cerebrospinal fluid (46), ear swabs (28), other body fluids (30), pus (366), sputum (10), stool (18), tracheal aspirate (6), and urine (469). Of all GNB isolates, 233 and 196 isolates were recovered from specimens collected from male and female patients respectively.

**Fig 1:**
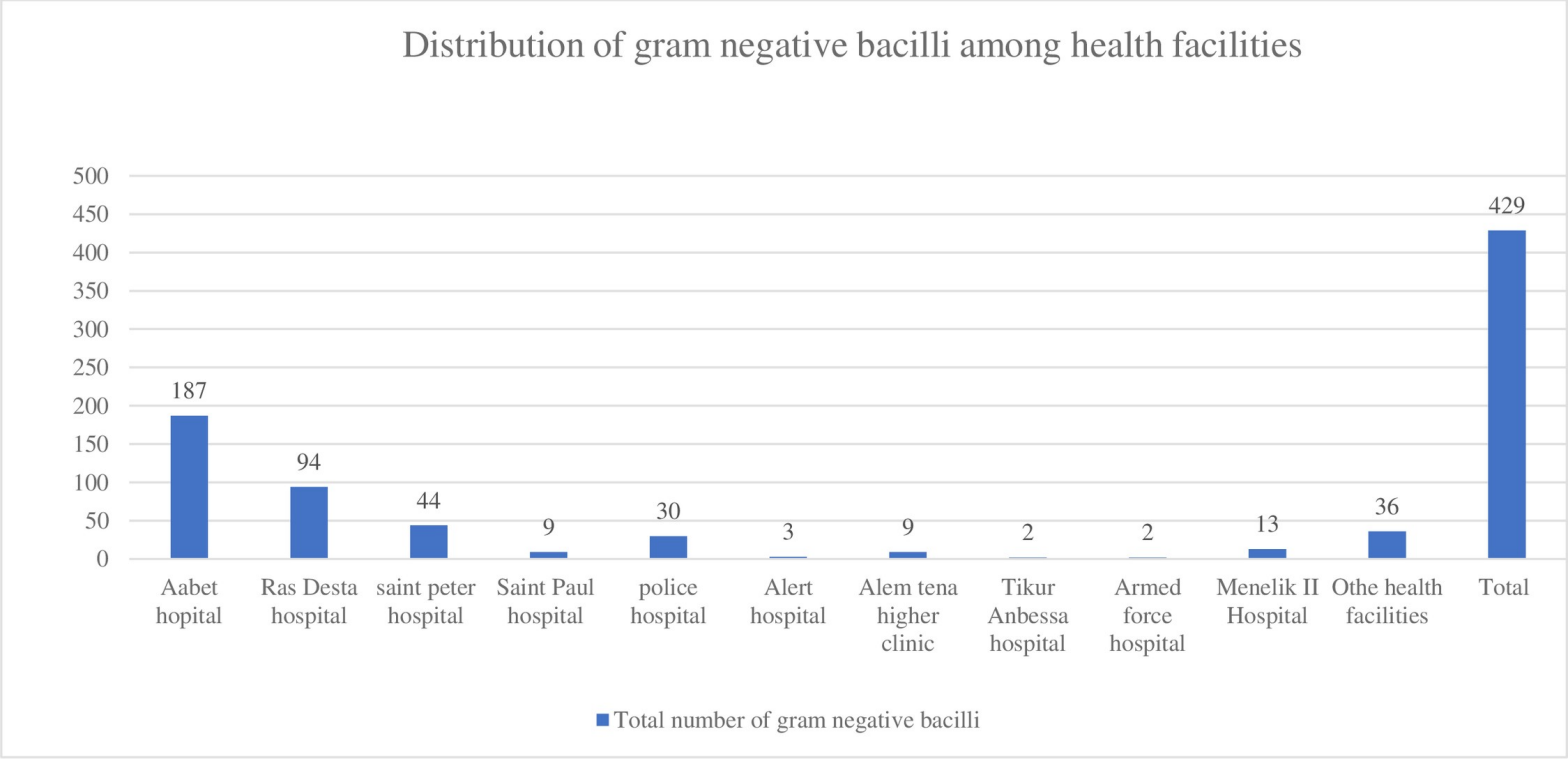
Distribution of gram-negative bacilli among health facilities.

Most of the isolates came from specimens referred from Aabet Hospital (187), Ras Desta Hospital (94), and Saint Peter’s Specialized Hospital (44). The distribution of GNB among health facilities is summarized in Fig 1.

**Fig 2:**
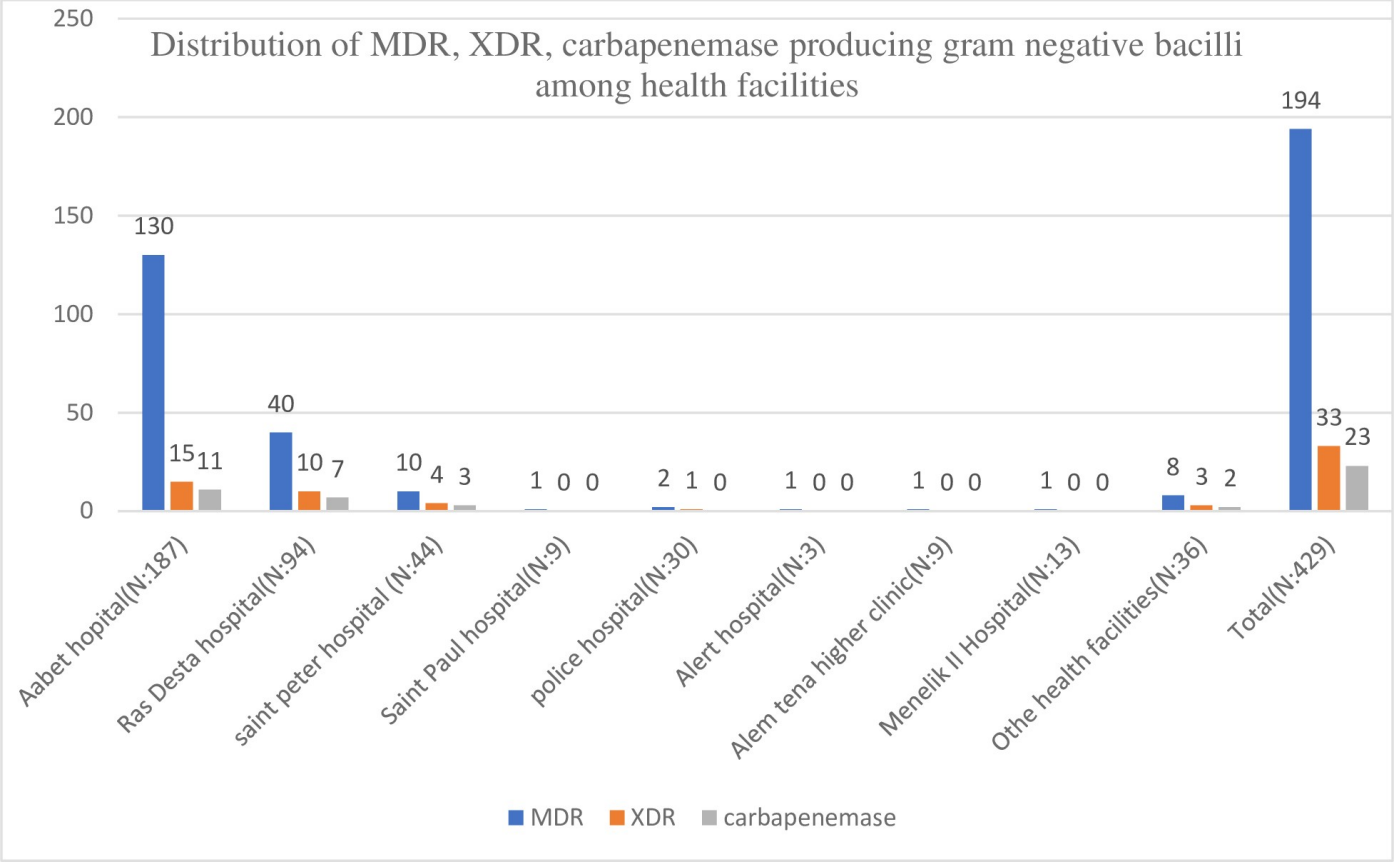
Distribution of MDR, XDR, carbapenemase-producing gram-negative bacilli among health facilities.

The highest number of MDR, XDR, and carbapenemase-producing isolates were recovered from specimens referred from Aabet Hospital, Ras Desta Hospital, and Saint Peter’s Specialized Hospital. One hundred eighty-seven (187) GNB were recovered from Aabet Hospital specimens of which 130 (69.5%), 15 (8%), and 11 (5.9%) were MDR, XDR, and carbapenemase producers respectively. Ninety-four (94) GNB were recovered from Ras Desta Hospital specimens of which 40 (42.5%), 10 (10.6%), and 7 (7.4%) were MDR, XDR, and carbapenemase producers, respectively. The distribution of MDR, XDR, and carbapenemase-producing isolates among health facilities is summarized in Fig 2.

### The prevalence of XDR gram-negative bacilli

Out of 429 GNB clinical isolates analyzed [33, 7.7%], were XDR; out of 33 XDR gram-negative isolates, *Acinetobacter* species were the predominant isolates 32.4% [24/74]. The remaining 9 XDR isolates were as follows; *K*. *pneumoniae* 2.7% [3/109], *Proteus mirabilis* 23.5% [4/17], *E*. *cloacae* 9% [1/11], and *P*. *aeruginosa* 2.7% [1/36] as summarized in Table 1. Of 33 XDR isolates [N = 21 or 63.64%] were isolated from intensive care units. The highest number of XDR gram-negative bacilli were isolated from urine samples [N = 16 or 48.48%]. The distribution of XDR isolates among specimen types, health facility wards and specimen sources is summarized in Table 1.

**Table 1:** Distribution of extremely drug-resistant gram-negative bacilli.

Distribution of XDR GNB			XDR GNB from different units			XDR GNB based on specimen sources		
Organism	N	%	Ward	N	%	Specimen type	N	%
*Acinetobacter* species	24	72.73	Intensive care unit	21	63.64	Urine	16	48.48
*K*. *pneumoniae*	3	9.09	Burn unit	2	6.06	Blood	7	21.21
*P*. *mirabilis*	4	12.12	Orthopedics	3	9.09	Sputum	1	3.03
*E*. *cloacae*	1	3.03	Unknown	6	18.18	CSF	1	3.03
*P*. *aeruginosa*	1	3.03	Emergency	1	3.03	pus	8	24.24
Total	33	100	Total	33	100	Total	33	100

XDR = Extremely drug-resistant; GNB = Gram-negative bacilli; N = Number; % = percentage

### The prevalence of multi-drug resistance and carbapenemase-producing GNB

Of 429 isolates, 194 were MDR GNB isolates. The most frequently isolated MDR organism was *K*. *pneumoniae* [N = 80 or 73.4%] followed by *Acinetobacter* species [N = 52 or 70.3%], and *E*. *coli* [N = 36 or 23.6%]. The MDR GNB are summarized in Table 2.

**Table 2:** Prevalence of multidrug resistance and extremely drug resistant, and carbapenemase-producing GNB against 8 antimicrobial classes.

Organisms	R_0_	R_1_	R_2_	R_3_	R_4_	R_5_	R_6_	R_7_	R_8_	N% MDR	N% XDR	N% Carbapenemase	N% MBL	N% Serine	N% KPC
***E*. *coli (N = 152)***	3	52	61	20	14	2	0	0	0	36(23.7)	0(0)	2(1.3)	1(0.7)	1(0.7)	1(0.7)
***K*. *pneumoniae (N = 109)***	0	0	36	51	10	9	0	1	2	80(73.4)	3(2.8)	9(8.3)	5(4.6)	4(3.7)	1(0.9)
***K*. *oxytoca (N = 10)***	0	0	9	1	0	0	0	0	0	1(10)	0(0)	0(0)	0(0)	0(0)	0(0)
***K*. *ozaenae (N = 11)***	0	0	2	4	5	0	0	0	0	2(18.2)	0(0)	2(18.2)	0(0)	2(18.2)	0(0)
***E*. *cloacae (N = 11)***	0	1	6	1	2	0	0	0	1	4(36.4)	1(9.1)	0(0)	0(0)	0(0)	0(0)
***P*. *mirabilis (N = 17)***	0	1	10	1	1	0	0	2	2	6(35.3)	4(23.5)	1(5.9)	1(5.9)	0(0)	0(0)
***P*. *vulgaris (N = 3)***	0	0	1	1	1	0	0	0	0	2(66.7)	0(0)	0(0)	0(0)	0(0)	0(0)
***M*. *morganii (N = 6)***	0	0	5	0	1	0	0	0	0	1(16.7)	0(0)	0(0)	0(0)	0(0)	0(0)
***Acinetobacter species (N = 74)***	0	0	22	0	3	5	20	10	14	52(70.3)	24(32.4)	5(6.8)	2(2.7)	3(4.1)	0(0)
***P*. *aeruginosa (N = 36)***	0	3	23	5	4	0	0	0	1	10(27.8)	1(2.8)	4(11.1)	1(2.8)	3(8.4)	1(2.8)
**Total (N = 429)**	3	57	175	84	41	16	20	13	20	194(45.2)	33(7.7)	23(5.4)	10(2.3)	13(3)	3(0.7)

Abbreviations: R_0_-R_8_: No resistance to antimicrobial class to Resistant to eight antimicrobial classes; MDR-Multidrug resistant, XDR-extensively drug-resistant, MBL-metallo-β-lactamase, KPC—*Klebsiella pneumoniae* carbapenemase, N-Number, %-percentage.

Of 429 isolates, 15.4% (66/429) isolates were non-susceptible to either meropenem or imipenem. Carbapenem non-susceptible isolates were considered candidates for carbapenemase screening. Of 66 isolates screened for carbapenemase, 34.8% (23/66) were carbapenemase enzyme producers (Table 2). Ten of twenty-three (10/23) carbapenemase-positive organisms were metallo-B-lactamase (MBL) producers. Thirteen of twenty-three (13/23) isolates were serine carbapenemase producers. Three of thirteen (3/13) serine carbapenemase-positive organisms were *Klebsiella pneumoniae* carbapenemase (KPC) producers. Of 10 metallo-B-lactamase positive isolates, 5 (50%) were *K*. *pneumoniae*, and the remaining testing results are summarized in Table 2. The 3 KPC isolates were *E*. *coli*, *K*. *pneumoniae*, and *P*. *aeruginosa* (Table 2).

#### The prevalence of multi-drug resistant and extremely drug resistant, and carbapenemase-producing GNB

Of 194 MDR GNB isolates, 45% were isolated from patients admitted to the intensive care unit, 83.4% were isolated from patients previously exposed to different antimicrobial agents, 28% were isolated from patients under mechanical ventilation and/or urinary catheterization, and 34.7% were isolated from patients with hospital-acquired (HAI) pneumonia (Table 3).

**Table 3:** Univariate analysis of MDR GNB infection.

Risk factors	MDR GNB		Non-MDR GNB			
	(N = 194)	%	(N = 235)	%	OR (95% CI)	P-value
Admission to an intensive care unit (N = 193)	(N = 134)	69.4	(N = 59)	30.6	2.75 (1.92–3.95)	0.001
Previous exposure to antimicrobials (N = 358)	(N = 169)	47.2	(N = 189)	52.8	2.79 (0.97–1.65)	0.069
Sepsis of different focus (N = 84)	(N = 75)	89.3	(N = 9)	10.7	15.83(7.66–32.72)	0.001
Mechanical ventilation and urinary catheterization (N = 120)	(N = 81)	67.5	(N = 39)	32.5	3.60 (2.3–5.6)	<0.001
Recurrent urinary tract infection (N = 88)	(N = 57)	64.8	(N = 31)	35.2	2.75(1.68–4.46)	<0.001
Hospital-acquired infection and hospital-acquired pneumonia(N = 149)	(N = 93)	62.4	(N = 56)	37.6	2.94 (1.95–4.44)	<0.001

MDR- Multidrug resistant, GNB—Gram-negative bacilli, OR—odds ratio, CI—confidence interval, N- Number, %-percentage

### Risk factors for acquiring multidrug resistant infections

Admission to an intensive care unit (OR:2.75, 95% CI: 1.92–3.95, P value: 0.001), mechanical ventilation as source of infection and/or urinary catheterization as source of infection (OR:3.6, 95% CI:2.3–5.6, P value:<0.001), HAI and/or HAI pneumonia as a source of infection (OR:2.94, 95% CI: 1.95–4.44, P value:<0.001), and recurrent urinary tract infection (OR:2.75, 95% CI:1.68–4.46, P value: <0.001), and sepsis originating from different focus (OR:15.83, 95% CI: 7.66–32.72, P value:0.001) were significantly associated with acquiring multidrug resistant gram-negative bacilli. (Table 3).

#### Univariate analysis of infections caused by MDR gram negative bacilli

Of 23 carbapenemase-positive organisms, 56.5% (13/23), 26.1% (6/23), 8.7% (2/23), 8.7% (2/23) were isolated from urine, pus, blood, and tracheal aspirate respectively (Table 4). Of 23 carbapenemase-positive organisms, 82.6% (19/23) and 17.4% (4/23) were isolated from the patients admitted to the intensive care unit and unknown ward respectively (Table 4). In this study the prevalence of carbapenem-nonsusceptible and carbapenemase-producing GNB is 15.4% (66/429) and 5.4% (23/429) respectively (Table 4).

**Table 4:** Distribution of carbapenemase among wards and specimen sources.

Specimen types	Carbapenemase			Wards	Carbapenemase		
	Positive	Negative	Total		Positive	Negative	Total
Blood	2	7	9	Emergency	0	6	6
Pleural fluid	0	1	1	ICU	19	22	41
Pus	6	11	17	Inpatient	0	3	3
CSF	0	2	2	outpatient	0	2	2
Tracheal aspirate	2	3	5	Unspecified	4	10	14
Tissue	0	1	1	**Total**	**23**	**43**	**66**
Urine	13	18	31				
**Total**	**23**	**43**	**66**				

ICU-Intensive care unit, CSF-Cerebrospinal fluid

### Distribution of carbapenemase among wards and specimen sources

#### Phenotypic antimicrobial resistance patterns of Enterobacterales

Of 429 gram-negative isolates, 293 were the Enterobacterales family (*E*. *coli*, *K*. *pneumoniae*, *K*. *oxytoca*, *K*. *ozaenae*) (Table 2). The highest resistance was observed against ampicillin by *E*. *coli* [89.3%] (Table 5). *K*. *pneumoniae*, *K*. *oxytoca*, and *K*. *ozaenae* are intrinsically resistant to ampicillin hence not tested against them [30].

**Table 5:** Antibiotic susceptibility pattern of gram-negative bacilli against different antimicrobial classes.

		*E*. *coli*	*K*. *pneumoniae*	*K*. *oxytoca*	*K*. *ozaenae*	*E*. *cloacae*	*P*. *mirabilis*	*P*. *vulgaris*	*M*. *morganii*	*Acinetobacter species*	*P*. *aeruginosa*
Ampicillin & β-lactam combinations	AMP %R	89.3	NA*	NA*	NA*	NA*	100	NA*	NA*	NA*	NA*
	AMC %R	33.8	64.4	33.3	87.5	NA*	38	0	NA*	NA*	NA*
	TPZ %R	13.3	33.3	14.3	66.7	42.9	30	0	17	85.4	12.5
1st and 2nd generation cephalosporins	CZ %R	60.4	85.7	57.1	100	NA*	80	NA*	NA*	NA*	NA*
	CXM %R	64.1	91.4	60	100	66.7	71	NA*	NA*	NA*	NA*
Extended-spectrum cephalosporins	CRO %R	63.8	88.7	50	100	100	69	0	67	94.7	NA*
	CTX %R	68.6	82.8	60	100	25	68	0	67	95.7	NA*
	CAZ %R	65	81.1	33.3	100	71.4	83	0	0	93	14.3
	FEP %R	55.7	81.8	40	85.7	75	89	0	0	86.1	21.7
Carbapenems	IPM %R	0	6.2	0	50	50	20	0	0	29.4	0
	MEM %R	2.2	5.2	0	20	9.1	14	0	25	48.1	19.4
Folate pathway antagonists	SXT %R	76.7	82.7	66.7	100	81.8	88	66.7	100	68.4	NA*
Quinolone and Fluoroquinolone	NA %R	83.3	80	NA*	NA*	0	100	0	100	NA*	NA*
	CIP %R	62.9	62.3	25	87.5	66.7	71	50	60	77.6	21.9
Tetracycline	TET %R	84.8	60	66.7	60	50	NA*	NA*	0	92.9	0
Nitrofurantoin	FM %R	3.1	46.4	33.3	75	0	NA*	NA*	NA*	NA*	NA*
Aminoglycosides	GEN %R	20.5	71.2	0	66.7	60	25	0	33	72.7	0
	TOB %R	33.6	37.2	25	72.7	57.1	63	0	25	54.3	17.2
	AMK %R	3.1	11.1	0	12.5	0	46	0	0	29.5	4.3

Abbreviations: AMP-Ampicillin, AMC-Amoxicillin-clavulanate, TZP-Piperacillin-tazobactam, CZ-Cefazolin, CXM-Cefuroxime, CRO-Ceftriaxone, CTX-Cefotaxime, CAZ-Ceftazidime, FEP-Cefepime, IPM-Imipenem, MEM-Meropenem, SXT-Trimethoprim-sulfamethoxazole, NA- Nalidixic acid, CIP-Ciprofloxacin, TET-Tetracycline, FM-Nitrofurantoin, GEN-Gentamycin, TOB-Tobramycin, AMK-Amikacin, %R-Percent resistant, NA*- Not Applicable, 0-Zero resistance.

*K*. *ozaenae* showed 100% resistance to first, second, and third generation cephalosporins as well as trimethoprim-sulfamethoxazole and *Enterobacter cloacae* demonstrated 100% resistance to ceftriaxone. The overall resistance profile of *Enterobacterales* to extended-spectrum (3^rd^ and 4^th^ generation) cephalosporins ranges from ceftriaxone [67.7%], cefotaxime [73.6%], ceftazidime [73.6%], and cefepime [66.5%]. *E*. *coli* showed high resistance to extended-spectrum cephalosporins, ceftriaxone [63.8%], cefotaxime [68.6%], ceftazidime [65%] and cefepime [55.7%] (Table 5). *K*. *pneumoniae* showed high resistance to extended-spectrum cephalosporins; ceftriaxone [88.7%], cefotaxime [82.8%], ceftazidime [81.1%], and cefepime [81.8%].

*E*. *coli* demonstrated high resistance to ciprofloxacin [62.9%] and trimethoprim-sulfamethoxazole [76.7%]; similarly, *K*. *pneumoniae* showed high resistance to ciprofloxacin [62.3%] and trimethoprim-sulfamethoxazole [82.7%] (Table 5). The overall prevalence of *Enterobacterales* resistance to gentamycin, tobramycin, and amikacin was 41.9%, 37.5%, and 6.1% respectively. The overall prevalence of meropenem and imipenem resistance among the *Enterobacterales* was 4.2% and 6.5% respectively.

*Morganella morganii*, *P*. *mirabilis*, and *P*. *vulgaris* were reorganized into the *Morganellaceae* family [30]. A total of 26 *Morganellaceae* isolates were identified during the study period (Table 2). *M*. *morganii* and *P*. *vulgaris* are intrinsically resistant to ampicillin, cefazolin, and cefuroxime and were not reported in Table 5. *M*. *morganii*, *P*. *mirabilis*, and *P*. *vulgaris* intrinsically resistant to antimicrobials (Table 5) and identified as Not Applicable/NA.

*P*. *mirabilis* showed 100% resistance to ampicillin, nalidixic acid, and trimethoprim-sulfamethoxazole; similarly, *M*. *morganii* demonstrated 100% resistance to nalidixic acid and trimethoprim-sulfamethoxazole. *Morganellaceae* showed high resistance percentage to ceftriaxone [80.8%], cefotaxime [61%], ceftazidime [57.7%], and cefepime [63.6%]. The percentages of meropenem and imipenem resistance were 14% and 14.3% respectively (Table 5). The antimicrobial susceptibility patterns of the *Morganellaceae* are summarized in Table 5. The percentage of *Morganellaceae* resistance among aminoglycosides ranges from 26.3% to 47.5% (Table 5).

### Phenotypic antimicrobial resistance patterns of *Acinetobacter* species

*Acinetobacter* species show the highest level of resistance to cefotaxime [95.7%], ceftriaxone [94.7], ceftazidime [93%], tetracycline [92.9%], and cefepime [86.1%]. *Acinetobacter* species showed lowest resistance to amikacin [29.4%], meropenem [48.1%], imipenem [29.4%] (Table 5).

### Phenotypic antimicrobial resistance patterns of *P*. *aeruginosa*

A total of 36 *P*. *aeruginosa* isolates were obtained during the study period. The highest percentage of resistance by *P*. *aeruginosa* was observed against ciprofloxacin [N = 32, 21.9%], cefepime [N = 32, 21.7%], and meropenem [N = 31, 19.4%] (Table 5).

## Discussion

The prevalence of XDR, MDR, carbapenemase-producing, and carbapenem-resistant GNB is increasing [5, 6, 37]. In our study, the prevalence of MDR, XDR, carbapenemase-producing, and carbapenem non-susceptible GNB is high.

### Extensively drug resistant gram-negative bacilli

The most frequently isolated XDR organism was *Acinetobacter* species 32.4% [24/74] which disagree with the study findings of Beyene et al (*E*. *coli* 18.1% was the dominant XDR GNB followed by *K*. *pneumoniae* 11.1% [37] and Gashaw et al (*Klebsiella* species 43.3% was the dominant XDR GNB) [38]. The difference could be attributed to geographical differences, the number of samples studied, or the types of gram-negative bacteria considered. In the present study, the highest number of XDR organisms were recovered from urine samples 48.48% [16/33] and patients admitted to an intensive care unit 63.64% [21/33].

In the present study, the prevalence of XDR gram-negative bacilli was 7.7% [33/429], which is slightly lower than a study at Ethiopian Public Health Institute, Ethiopia by Beyene et al 8.8% [37]. This variation might be due to the investigators analyzing only Enterobacterales and is much lower than the findings from the study at Jimma, Ethiopia by Gashaw et al 41.3% [38], Arsho Advanced Medical Laboratory, Addis Ababa, Ethiopia by Bitew et al 34.3% [39], and the Tertiary Care Hospital, Pakistan by Abbas et al 64% [40]. The variation might be due to geographic location, the technique utilized, XDR definition, types of organism, etc.

### Carbapenemase producing gram negative bacilli

In our study, the prevalence of carbapenemase-producing gram-negative bacilli was 5.4%, which is higher than the prevalence of study conducted at the University of Gondar, Ethiopia by Eshetie et al at 2.72% [41] and the Ethiopian Public health Institute by Beyene et al at 2% [37]. However, our result was lower than the result of studies conducted at Tikur Anbessa Specialized Hospital, Ethiopia by Melese et al with 12.12% [42], Three Hospitals in Amhara region, Ethiopia by Moges et al 15.7% [43], Felegehiwot Hospital, Ethiopia by Moges et al 16.2% [44], Sidama, Ethiopia by Alemayehu et al 9% [45], Mulago National Referral Hospital, Uganda by Okoche et al 22.4% [46], and data from laboratories in Spain by Lopez-Hernandez et al 62% [47]. The variation might be due to the method utilized, i.e. the modified carbapenem inactivation method was utilized in our investigation unlike other investigators who used the modified Hodge test, the number of bacterial isolates analyzed, geographic location. However, our study findings are in line with a prospective cross-sectional study conducted at Felegehiwot Hospital, Ethiopia by Alebel et al 5.2% [48]. The dominant carbapenemase-producing gram-negative bacilli were *Klebsiella pneumoniae* 8.3% [9/109] followed by *Acinetobacter* species 6.8% [5/74]. Our study results marginally coincides with the findings of the following researchers which demonstrated the dominant prevalence of carbapenemase-producing *Klebsiella pneumoniae*: Beyene et al *Klebsiella pneumoniae* 5.6% [37], Melese et al *Klebsiella pneumoniae* 10.5% [42], Moges et al *Klebsiella pneumoniae* 5.8% [43], Moges et al *Klebsiella pneumoniae* 10.1% [44], Lopez-Hernandez *Klebsiella pneumoniae* 45% [47]. The present study disagrees with the findings by Okoche et al, which showed *E*. *coli* as the highest carbapenemase-producing organisms [46], The discrepancy could be explained by the fact that they utilized boronic acid-based inhibition, a modified Hodge test and EDTA double combination disk test, the number of samples analyzed, and a different geographical location.

The highest number of carbapenemase producing isolates were recovered from urine samples 13/23 (56.5%), which strongly disagrees with the results of study conducted by Moges et al in which the highest number of carbapenemase producing isolates was recovered from blood samples 22/24 (91.6%) [43].

### Multidrug resistant gram-negative bacilli

The prevalence of MDR was 45.2% which is much lower than the study conducted at the Ethiopian Public Health Institute by Beyene et al 94.5% [37], three referral hospitals, Ethiopia by Moges et al 85.8% [43], Felegehiwot Hospital, Ethiopia by Moges et al 80% and by Alebel et al 81.1% [44, 48], Addis Ababa, Ethiopia by Teklu et al 68.3% [49], northern Iran by Hemmati 62.8% [50], and Jimma Medical Center, Ethiopia by Biset et al 56.67% [51]. The variation might be due to the study population, the number of isolates assessed or the test method utilized. However, our study findings are in line with the study conducted at Arsho Advanced Medical Laboratory, Addis Ababa, Ethiopia by Bitew et al 42.1% [39]. The dominant MDR isolates were *K*. *pneumoniae* 80 (73.4%); however our results disagree with the study findings of Beyene et al in which *E*. *coli* (99.3%) is the dominant one followed by *K*. *pneumoniae* (90.3%) [37]. The observed variation could be attributable to the study period and proportion of bacterial isolates, Alebel et al in which *Acinetobacter species* (100%), *P*. *aeruginosa* (100%), *Citrobacter* species (100%), and *Enterobacter* cloacae (100%) were the dominant MDR isolates [48]. This variation could be attributable to fewer samples evaluated, samples were taken from intensive care unit patients, or that the study was conducted in a different geographic location and Biset et al [51] the difference might be since they only analyzed urine samples among pregnant women. Likewise, the following researchers reported the dominant prevalence of MDR *Klebsiella* species Moges et al *Klebsiella* species (30.6%) [43], Moges et al. *K*. *pneumoniae* (53.3%) [44], and Teklu et al *K*. *pneumoniae* (83.5%) [49].

### Carbapenem-resistant gram-negative bacilli

The prevalence of carbapenem-resistant gram-negative bacilli was 10.7% (46/429), in which *Acinetobacter* species account for about 39% of carbapenem-resistant isolates. This finding contradicts the study findings of the following researchers: Beyene et al 1.7% [37], Alebel et al 21% [48], Teklu et al 5.2% [49], Gashaw et al 25% [38], Melese et al 12.2% [42], in *K*. *pneumoniae* accounts for 100%, 40%, 50%, 36%, and 75% of the gram-negative bacilli isolates respectively. The observed difference could be attributed to different gram-negative bacteria analyzed, as most of them only included *Enterobacterales*, the techniques used, geographical location, and so on.

### Resistance patterns of Gram-negative bacilli to extended spectrum cephalosporins

The total resistance profile of Enterobacterales to extended-spectrum cephalosporins ranges from 57.7% to 80.8%, which marginally agrees with results of study conducted by Beyene et al 73.5% to 73.9% [37], and by Breurec S et al 79.7% [52] but higher than the study findings of Teklu et al (60.3% to 62.2%) [49], Gashe F et al (56.5% to 60.1%) [53] and Moges et al (60.3% to 66%, in 2021) [43] lower than the study finding by Moges et al (87.2% to 96.6%, in 2019) [44]. The geographical location, study time, and quantity of samples analyzed could all be factors in the reported discrepancy. Extended spectrum cephalosporin resistance was found in 17.5% of clinical Enterobacterales isolates studied in North America and Europe between 2016 and 2018 [54]; the observed discrepancy could be attributed to the study’s large geographical scope, infection control techniques used in those settings, the number of samples tested, and other factors.

## Conclusion and recommendations

This study revealed an alarming level of AMR, with a high prevalence of multidrug-resistant, extremely drug-resistant, carbapenemase-producing gram-negative bacteria, particularly among intensive care unit patients at the health facility level. These findings point to a scenario in which the clinical management of infected patients becomes increasingly difficult and necessitates the use of last-resort antimicrobials, likely exacerbating the magnitude of the global AMR crisis. This necessitates a robust antimicrobial resistance monitoring and infection prevention and control program at these institutions.

### Limitations of the study

The responsible genes for carbapenemase production were not genotypically assessed. Further, patient clinical impact was not assessed. Tigecycline, colistin, and fosfomycin were not available and accordingly not used for AST.

## Supporting information

S1 Data Patient’s demographic and MDR data. (XLSX)

S2 Data Patient’s demographic, carbapenemase and extremely drug resistance data. (XLSX)
